# Coronary Computed Tomography Angiography and Abdominal Aortic Calcification Screening among High-Risk Living Kidney Donors

**DOI:** 10.3390/jcm12134541

**Published:** 2023-07-07

**Authors:** Keren Skalsky, Arthur Shiyovich, Nir Hochwald, Amos Levi, Lutof Zreik, Shlomit Tamir, Gideon Shafir, Anat Briger, Ruth Rahamimov, Ran Kornowski, Ashraf Hamdan

**Affiliations:** 1 Department of Cardiology, Rabin Medical Center, Petach-Tikva 4941492, Israel; 2 Faculty of Medicine, Tel Aviv University, Tel Aviv 6997801, Israel; lutof.zreik@gmail.com (L.Z.);; 3 Department of Imaging, Rabin Medical Center, Petach-Tikva 4941492, Israel; 4 Department of Organ Transplantation, Rabin Medical Center, Petach-Tikva 4941492, Israel; 5 Department of Nephrology, Rabin Medical Center, Petach-Tikva 4941492, Israel

**Keywords:** living kidney donors, cardiovascular risk, coronary CTA

## Abstract

Background: A unique scanning protocol combining coronary computed tomography angiography (CTA) with routine abdominal CTA is being used at the Rabin Medical Center as a method of screening high-risk candidates for living kidney donation. We aim to evaluate the potential impact of coronary CTA on the decision regarding eligibility for kidney donation and its correlation with abdominal aortic calcification (AAC). Methods: CCTA and abdominal CTA results of potential living kidney donors evaluated for donation between September 2020 and November 2021 were retrieved. A retrospective analysis of the abdominal CTA was used to calculate the AAC. Patients’ demographic, clinical, and imaging data were collected from the electronic files, as well as the final decision regarding eligibility for donation. Results: A total of 62 potential kidney donors were evaluated for donation using the combined scan. The mean age was 53.8 years, with male predominance (59.7%). Significant coronary artery stenosis (≥70% luminal stenosis) was present in two patients (3.2%), whereas five patients (8%) had moderate stenosis (50–69%). Thirteen patients (21%) were disqualified from donation due to moderate-to-significant coronary artery disease or abdominal atherosclerosis. The correlation between the coronary artery calcium score and the AAC was found to be positive, with a Pearson correlation coefficient of 0.88 (*p* < 0.001). Conclusions: The use of coronary CTA in the evaluation of potential kidney donors may has a potential impact on the decision regarding eligibility for donation. A high correlation between the coronary artery calcium score and the AAC was found.

## 1. Introduction

Living kidney donor transplantation plays a substantial role in the field of organ transplantation, comprising approximately 30% of all kidney transplantations carried out annually in the United States [1]. In Israel, this procedure accounts for an even higher percentage, with over 60% of kidney transplantations being attributed to living kidney donors. However, despite the positive impact of living kidney donation, long-term studies have indicated that donors face an increased risk of developing end-stage kidney disease, all-cause mortality, cardiovascular mortality [2], and ischemic heart disease [3].

Given these potential risks, it is important to thoroughly evaluate and assess potential kidney donor candidates. The evaluation process serves two essential purposes: determining their suitability for donation and identifying any potential future health issues that may arise. Special attention should be given to candidates with cardiovascular risk factors during this assessment. The current guidelines recommend pre-donation assessment of the abdominal vasculature anatomy with computed tomography, as well as cardiovascular risk assessment according to the donor’s age, blood tests, and the presence of cardiovascular risk factors [1,4,5].

Invasive coronary angiography is considered to be the reference standard for diagnosing obstructive coronary artery disease due to its high diagnostic performance and the long-time experience with this method [6]. Nevertheless, in only 38–40% of patients undergoing invasive coronary angiography is obstructive coronary artery disease found [7]. False positive results by traditional tests, such as exercise electrocardiograms, stress echocardiography, or myocardial scintigraphy are potential reasons for the low diagnostic yield of this invasive procedure [6]. Over the last few years, coronary computed tomography angiography (CTA) has been developed into a noninvasive alternative to the invasive strategy with excellent diagnostic accuracy for ruling out coronary artery disease [8], and even for prediction of future cardiovascular events [9]. Based on the previous guidelines, coronary CTA was the recommended imaging modality for the evaluation of patients with chronic coronary syndrome (class I recommendation, level of evidence C) [10]. The test has an advantage over functional tests in its high sensitivity, specificity, and diagnostic performance for the detection or exclusion of coronary artery disease, both obstructive and non-obstructive. The test enables the targeting of preventative therapies at all stages of coronary stenosis, which has the potential to impact future outcomes [10,11,12,13,14]. Furthermore, the coronary calcium score (CAC) is a potent determinant of risk since it serves as an indicator of atherosclerotic condition, encompassing both recognized and undisclosed risk factors [15]. Recent findings have indicated that abdominal aortic calcification (AAC) could also be considered as a risk modifier for cardiovascular disease [16], given its comparable ability to predict cardiovascular disease when compared to CAC [17].

Since September 2020, a unique scanning protocol combining CTA of the abdominal aorta and renal arteries along with coronary CTA is being used at the Rabin Medical Center in potential living kidney donors with cardiovascular risk factors for risk stratification. In the present study, we aimed to evaluate the potential impact of coronary CTA on the decision regarding eligibility for kidney donation, as well as to exam the correlation between CAC and AAC in a retrospective analysis of consecutive kidney donor candidates.

## 2. Materials and Methods

### 2.1. Patients/Population

Potential kidney donors older than 50 years, as well as those with a family history of ischemic heart disease or more than one cardiovascular risk factor (essential hypertension, diabetes mellitus, dyslipidemia, active smoking, and family history of ischemic heart disease) were evaluated in the cardiology clinic and were sent to coronary CTA. All potential donors underwent abdominal CTA to assess their renal and peripheral vascular anatomy, and for those who were send for coronary CTA, both scans were performed together. The imaging results were used as risk modifiers for decision making.

### 2.2. Design

A retrospective registry of potential living kidney donors who underwent coronary CTA between the years 2000 and 2021 at the Rabin Medical Center were included. Their medical histories, including patient demographics, blood pressure measurements, creatinine level, lipid profile, fasting glucose level, diabetes status, family history of coronary artery disease, and smoking status were recorded. The coronary CTA results were then retrieved, including the coronary CAC score and the degree of coronary luminal stenosis.

Coronary artery segments were assessed for the presence of stenosis using five predefined categories: no coronary artery disease (CAD) (0%), minimal CAD (1–24% stenosis), mild CAD (25–49% stenosis), moderate CAD (50–69% stenosis in any major vessels/branch), and significant CAD (≥50% stenosis of the left main (LM) or ≥70% in any major vessel/branch).

The coronary Artery Disease-Reporting and Data System (CAD-RADS) was used for calculations [18]. Quantification of the CAC was performed using the acceptable methods [19]. The CAC scores were categorized as 0, 1 to 99, 100 to 399, or ≥400 Hounsfield units.

The final decisions regarding potential eligibility for donation were made taken by a multidisciplinary transplantation team, which included a nephrologist, a cardiologist, and an imaging specialist. A retrospective analysis of the abdominal CT was used to calculate the AAC score and its correlation with the CAC score.

### 2.3. CTA Acquisition Protocol

CTA scans were performed on a 256-slice CT machine (iCT, Philips, Amsterdam, The Netherlands) or a dual-source machine (Siemens Somatom Force, DSCT, Munich, Germany). Philips scans included the following: (1) CAC scoring: non-contrast CT scan of the upper abdomen with a slice thickness of 2.5 mm. (2) Coronary CTA: data were acquired with a collimation of 96 × 0.625 mm and a gantry rotation time of 330 ms. The tube current was 485 mA at 100 kV, the pitch value was 0.2, and the scan direction was craniocaudal. Intravenous injection of 145 cc Omnipaque 350 (GE healthcare, Chicago, IL, USA) at a flow rate of 5 mL/s was followed by a 30 mL saline chase bolus (5 mL/s). The size of the intravenous cannula used was 20-gauge, equating to an inner diameter of about 0.62 mm (0.024 inches). Automated peak enhancement detection in the descending aorta was used to time of the scan. Data acquisition was automatically initiated at a threshold level of 100 Hounsfield units. Acquisition was performed during an inspiratory breath hold, while the electrocardiogram was recorded simultaneously to allow for retrospective ECG gating of the data. (3) The abdominal and pelvic scan was immediately performed after the CTA scan, and the data were reconstructed using a 2.5 mm slice thickness. Finally, a scout of the abdomen and pelvis was performed. Siemens scans included the following: CAC scoring; non-contrast scan of the upper abdomen reconstructed with a slice thickness of 3 mm; and gated cardiac CT reconstructed with a slice thickness of 0.6 mm after the intravenous injection of 70 cc Omnipaque 350 (GE healthcare), followed immediately by a scan of the abdomen and pelvis with a slice thickness of 1 mm using the Turbo Flash technique. Finally, a scout of the abdomen and pelvis was performed. The non-contrast scan of the upper abdomen was aimed to evaluate renal calculi; the late arterial phase of the abdomen and pelvis was aimed for evaluation of the renal vasculature, including the arteries and veins; and the delayed scout was performed in order to visualize the collecting systems for anatomical variants [20].

### 2.4. Image Reconstruction and Data Analysis

The 3-dimensional data set of the contrast-enhanced scan was reconstructed at the systolic (30% and 40% of the cardiac cycle) and diastolic phases (70%, 75%, and 80% of the cardiac cycle). The iterative reconstruction approach used for reconstruction of the coronary CTA data was based on an initial filter back-projection with a very sharp convolution algorithm containing all information from the initial raw data. Subsequent iterative processing loops were applied to the image volume to reduce the image noise while preserving the spatial resolution. For data analysis, the complete data set was transmitted to a dedicated CT workstation (Extended Brilliance Workspace, version 12; Philips) for postprocessing by an experienced cardiac imager. Depending on the vessels’ morphology, various postprocessing techniques were applied. In addition to an assessment of the original axial slices, curved multiplanar reconstructions and 3-dimensional, volume-rendered reconstructions were used for coronary artery evaluation.

### 2.5. Statistical Analysis

Continuous variables are reported as means (±SD), as well as medians and interquartile ranges, when appropriate. Categorical variables are described as n (%). Correlations were plotted using scatter plots with log transformation of both axes, then tested using the Pearson correlation test. All tests were 2-sided, and a value of *p* < 0.05 was considered to be significant. All analyses were performed using R (R-studio, V.4.0.0, Vienna, Austria).

## 3. Results

A total of 62 potential kidney donors were evaluated for donation, utilizing coronary CTA as the screening method, between September 2020 and November 2021. The mean age of the participants was 53.8 years, with male predominance (59.7%).

The average blood pressure obtained from the group was 122 over 70 mmHg, while the low-density lipoprotein (LDL) level was found to be 109 mg/dL, as indicated in Table 1. The most common indication for coronary CTA screening was age above 50 years, with only 8% of the patients screened due to other reasons, including smoking status and family history of ischemic heart disease. Nitroglycerine was used in every patient, and beta blockers were administrated whenever than heart rate rose to more than 65 beats per minute; this was used in 35% of causes. No other medications were used.

**Figure 1:**
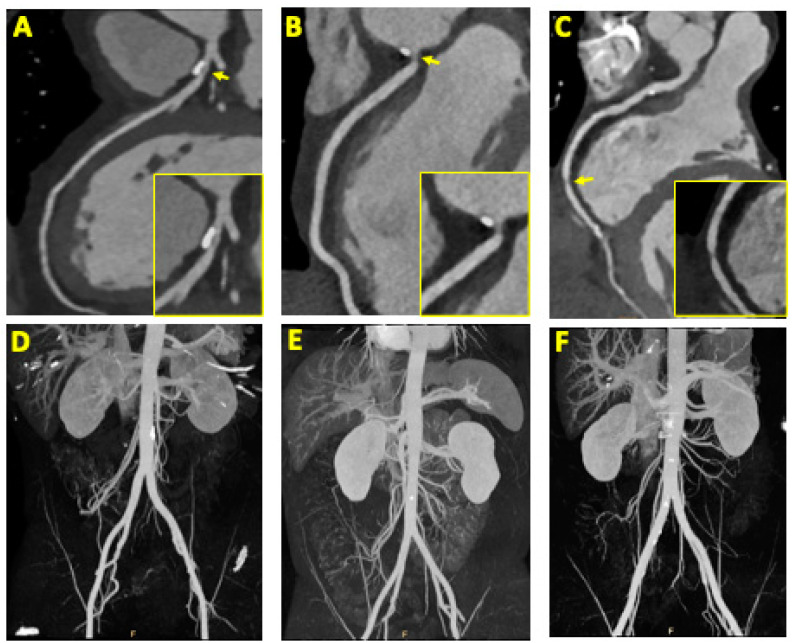
Representative examples of mild, moderate, and significant coronary artery stenosis with the corresponding aortoiliac arteries: Multiplanar and straightened reconstruction representing mild stenosis (25–49% luminal stenosis) of the LAD (**A**), moderate stenosis (50–69% luminal stenosis) of the RCA (**B**), and significant stenosis (≥70% luminal stenosis) of the RCA (**C**). The corresponding maximal intensity projections of the aortoiliac artery are also presented (**D**–**F**).

Quantification of the CAC of the potential donors provided insights into the extent of arterial calcification. In total, 47% of the participants displayed calcium scores of 0, indicating the absence of calcification, while 24.2% fell within the range of 1–99, signifying minimal calcification. In contrast, 47% exhibited scores between 100–399, indicating a moderate degree of calcification, and a mere 1.6% received scores exceeding 400, which denoted a high level of calcification. CAD RADS was 2, 3, 4A, or N in 10%, 5%, 3%, and 2% of the patients, respectively. Significant stenosis was present in only 3.2% of the patients (2 patients), whereas 8% (5 patients) had at least moderate stenosis and 19.3% (12 patients) had evidence of at least mild (25–49%) stenosis. Minimal coronary artery stenosis was recorded in 24.1% of the patients (15 patients). Figure 1A–C provide visual representations of these findings. The most affected vessel was the left anterior descending coronary artery (LAD), which showed at least mild stenosis in eight patients. Following closely was the circumflex artery (CX), which exhibited signs of at least mild stenosis in seven patients. The right coronary artery (RCA) and the left main coronary artery were affected in four and one patient(s), respectively. Notably, one patient with severe mid LAD stenosis experienced clinical angina, indicative of the symptomatic nature of his coronary artery disease, and was referred for invasive coronary angiography.

The seven patients with moderate or severe coronary artery disease were disqualified from donation to prevent further increases in their future cardiovascular risk. Significant abdominal atherosclerosis was the second reason for disqualification in six patients. Another fifteen patients were deemed ineligible for donation for other reasons, with the most common being unfavorable renal artery anatomy and the presence of malignancies. Three potential donors exhibited duplicated renal arteries, and one individual presented with polycystic kidneys. The malignancies demonstrated a range of diagnoses in four potential donors, including renal adenoma, melanoma, chronic lymphocytic leukemia, and prostate cancer.

Among the remaining potential donors, those with mild or minimal stenosis in their coronary arteries were considered to have an increased cardiovascular risk.

The multidisciplinary transplant team carefully evaluated each individual’s overall risk profile, taking into account the known cardiovascular risk factors and the results of the imaging tests. After thorough assessment and ensuring that the potential donors fully understood the associated risks, some of them were approved for donation. All potential donors with coronary atherosclerosis of any degree were guided to take preventive therapies, regardless of the decision regarding donation. The characteristics of the patients approved for donation compared to those found ineligible for donation are illustrated in Table 2. The triglyceride levels were higher among those not approved for donation, but no other significant differences were found between the groups.

**Figure 2:**
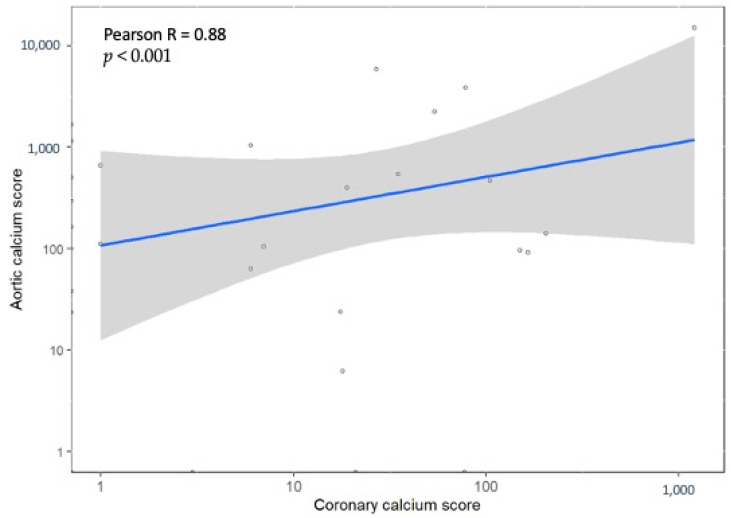
The correlation between the coronary calcium score and aortic calcium score. Pearson correlation coefficient was 0.88 (*p* < 0.001).

Data on both CAC and AAC were available for 50 potential donors. Among these individuals, 20 had calcium scores of zero in both CAC and AAC, indicating a relatively lower risk of atherosclerosis in both the coronary and abdominal arteries. For 12 patients, their calcium scores were zero in one of the tests but not in the other, suggesting some variability in the distribution of calcification. Finally, 18 patients exhibited positive calcium scores in both CAC and AAC, indicating the presence of calcified plaques in both the coronary and abdominal arteries. Upon analyzing the data, a strong and statistically significant correlation was observed between CAC and AAC, with a Pearson correlation coefficient of 0.88 and a *p*-value of less than 0.001. This finding indicates that the presence and extent of calcification in the coronary arteries tend to be associated with a similar pattern in the abdominal aorta. Figure 1A–F and Figure 2 visually represent the correlation and provide a graphical representation of the relationship between CAC and AAC in the studied group of potential kidney donors. The total radiation dose for the patients undergoing coronary and abdominal CTA, in both studies, was 17.5 ± 4.6 mSv (mean ± SD), with 7 mSv assigned to the cardiac scan. The amount of contrast agents did not change compared to that required for the abdominal scan. To note, furosemide was not used for the delayed scout, as the imaging of the renal collecting system was sufficient for all patients.

## 4. Discussion

Our study suggests two techniques for cardiovascular risk estimation in healthy potential kidney donors. The first technique involves the implementation of an extended CTA scan protocol, which offers a comprehensive evaluation of both the coronary and abdominal arteries, with limited exposure to radiation and contrast agents. This “one-stop shop” approach enables physicians to obtain detailed and reliable information about abdominal vasculature and future cardiovascular risk in the potential donor. The combined scan unmasked occult coronary artery disease in more than 19% of the potential kidney donors, which allowed for more accurate clinical judgments of both the patient and the physician to be made. Moreover, all of those patients with at least mild stenosis were guided to receive statin therapy, which has been proven to lower future cardiovascular risk in non-obstructive CAD [14]. The second technique involves utilizing AAC as a risk modifier during the selection process for kidney donors. Our study revealed that AAC can serve as a valuable indicator of underlying cardiovascular disease and can aid in making informed decisions regarding the eligibility of individuals as kidney donors.

The current guidelines recommend that lipid profile, smoking status, blood pressure, diabetes screening, and obesity would be part of the kidney donors’ assessment, although there are no uniform criteria for approval or disapproval for kidney donation. According to the AJKD’s Core Curriculum in Nephrology, published in 2018 [21], hypertension is controversial; patients with well-controlled hypertension and no end organ damage may be accepted for donation in some centers, while other centers will exclude any donors using antihypertensive medications. BMI cutoffs for donation also vary in different centers, and may vary between 30 and 35 kg/m^2^ [1]. The KDIGO guidelines suggest that candidates with well-controlled hypertension may be acceptable for donation, as well as older candidates with type 2 diabetes mellitus and well-controlled glycemia [4]. Detailed evaluations of the risk factors and their implications are discussed in each consensus document, but none of them consider the use of coronary CTA or AAC [1,4]. The only guidelines considering cardiologic evaluation and/or CAC and/or functional assessments in high-risk patients are the joint British Transplantation Society/Renal Association Guidelines [5]. In the absence of evidence, decisions are mostly based on expert opinion.

The advantage of coronary CTA over non-invasive functional tests lies in its ability to detect coronary artery stenosis even in its early stages. By identifying coronary artery stenosis early on, coronary CTA aids in improving risk stratification and informed decision-making for kidney donation. Not only does this information play a vital role in determining the suitability of individuals for kidney donation, it also holds immense potential for preventing long-term cardiovascular morbidity. Achieving strict control over cardiovascular risk factors, such as managing lipid profiles and increasing patient awareness, can be instrumental in averting the development of coronary artery disease. Various studies have emphasized the significance of coronary CTA in this regard, further highlighting its potential for long-term cardiovascular disease prevention [10,12,13,14,15]. Additionally, the presence of AAC on abdominal CTA serves as an additional source of valuable information. AAC has been proven to be a strong predictor of future cardiovascular events, surpassing the accuracy of the traditional Framingham risk score in asymptomatic individuals [22]. These cardiac scans necessitated mild additional radiation exposure, but did not involve any extra contrast exposure. These factors should be taken into consideration when evaluating the potential long-term advantages of coronary scans, the exact impact of which cannot be measured. The use of coronary and abdominal MRA might be an alternative; however, coronary MRA exhibits a lower diagnostic performance compared with coronary CTA, and is more time-consuming.

We suggest expanding the cardiovascular risk profile estimation of kidney donor by using the “one-stop shop” imaging technique, which includes the combined use of coronary and abdominal CTA along with the assessment of CAC and AAC scores. This test should be primarily limited to older candidates, specifically those above 50 years old, or it can be selectively employed for donors who exhibit other known cardiovascular risk factors. Further investigation is needed in order to characterize the potential donors that would benefit from each of these tests.

The main limitations of the study are its retrospective nature and the modest sample size. These factors render it prone to confounding. Additional larger-scale prospective studies are needed in order to establish more robust conclusions. These future investigations should aim to evaluate the combined scans’ efficacy in terms of long-term clinical outcomes following kidney donation, providing a clearer understanding of its practical utility and potential benefits for the donors.

## 5. Conclusions

The use of coronary CTA in combination with abdominal CTA in a “one-stop shop” for the evaluation of potential kidney donors could potentially influence the decision regarding their eligibility for donation. A strong correlation between the CAC score and the AAC was found.

## Figures and Tables

**Table 1:** Baseline characteristics of the potential living kidney donors.

	Potential Living Kidney Donors (*n* = 62)
Age (years)	53.8 ± 8.6
Female gender (%)	40.3
Weight (kg)	70.2 ± 9.8
Height (cm)	170.2 ± 9
BMI (kg/m^2^)	24.5 ± 2.8
Waist circumference (cm)	86.7 ± 9.9
Essential hypertension (%)	11.9
Systolic blood pressure (mmHg)	122 ± 12.5
Diastolic blood pressure (mmHg)	70 ± 5.9
Family history of ischemic heart disease (%)	23
Past or active smokers (%)	22
Hemoglobin A1c (%)	5.4 ± 4
Total cholesterol (mg/dL)	188.7 ± 37.6
LDL (mg/dL)	109 ± 30.3
HDL (mg/dL)	59.4 ± 16.8
Triglycerides (mg/dL)	107.5 ± 70.6
Creatinine level (mg/dL)	0.9 ± 0.2
Urea (mg/dL)	29.6 ± 5.8
Urine creatinine per 24 h (mg)	1456.8 ± 385.6
24 h urinary protein (mg)	7.1 ± 18.3
eGFR (mL/min/1.73 m^2^)	89.8 ± 13.4

Data are presented as mean ± standard deviation or as percentages. BMI—body mass index; LDL—low-density lipoprotein; HDL—high-density lipoprotein; eGFR—estimated glomerular filtration rate.

**Table 2:** Characteristics of patients approved for donation compared to those not approved.

	Potential Living Kidney Donors		
	Not Approved for Donation (*n* = 22)	Approved for Donation (*n* = 36)	*p* Value
Age (years)	55.7 ± 9.1	53.2 ± 7.6	0.28
Female gender (%)	37.5	44.1	0.79
BMI (kg/m^2^)	25.1 ± 2.5	23.9 ± 2.8	0.08
Waist circumference (cm)	89.9 ± 8.8	84.8 ± 10.4	0.07
Essential hypertension (%)	13	12.1	1
Systolic blood pressure (mmHg)	123 ± 11.7	120.8 ± 13.1	0.49
Diastolic blood pressure (mmHg)	70 ± 5.3	70.2 ± 6.1	0.88
Family history of ischemic heart disease (%)	16.7	29.4	0.35
Past or active smokers (%)	23	21	0.72
Hemoglobin A1c (%)	5.3 ± 0.4	5.4 ± 0.2	0.58
Total cholesterol (mg/dL)	191.5 ± 40.3	189.7 ± 36.2	0.86
LDL (mg/dL)	108.8 ± 31.5	111.2 ± 30.7	0.78
HDL (mg/dL)	55.8 ± 16.5	62.4 ± 16.5	0.14
Triglycerides (mg/dL)	137 ± 97.8	89.8 ± 37.2	0.03
Creatinine level (mg/dL)	0.9 ± 0.2	0.8 ± 0.2	0.27
Urea (mg/dL)	29.2 ± 6.2	30.3 ± 5.6	0.48
Urine creatinine per 24 h (mg)	1532.6 ± 379.2	1410.4 ± 392.1	0.25
24 h urinary protein (mg)	10.5 ± 29.7	5.2 ± 3.7	0.46
eGFR (mL/min/1.73 m^2^)	87.2 ± 13.9	91 ± 13.4	0.31

Data are presented as mean ± standard deviation or as percentages. BMI—body mass index; LDL—low-density lipoprotein; HDL—high-density lipoprotein; eGFR—estimated glomerular filtration rate.

## Data Availability

Data will be available for sharing upon request.
